# Stroma AReactive Invasion Front Areas (SARIFA)—A New Easily to Determine Biomarker in Colon Cancer—Results of a Retrospective Study

**DOI:** 10.3390/cancers13194880

**Published:** 2021-09-29

**Authors:** Benedikt Martin, Bianca Grosser, Lana Kempkens, Silvia Miller, Svenja Bauer, Christine Dhillon, Bettina Monika Banner, Eva-Maria Brendel, Éva Sipos, Dmytro Vlasenko, Gerhard Schenkirsch, Stefan Schiele, Gernot Müller, Bruno Märkl

**Affiliations:** 1 General Pathology and Molecular Diagnostics, Medical Faculty, University of Augsburg, 86156 Augsburg, Germany; bendikt.martin@uk-augsburg.de (B.M.); bianca.grosser@uk-augsburg.de (B.G.); lkempkens@web.de (L.K.); silvia.miller@uk-augsburg.de (S.M.); svenjabauer96@gmx.de (S.B.); christine.dhillon@uk-augsburg.de (C.D.); bettina-monika@gmx.de (B.M.B.); Eva-Maria.Brendel@uk-augsburg.de (E.-M.B.); eve.sipos@gmail.com (É.S.); 2 General, Visceral and Transplantation Surgery, University Hospital of Augsburg, 86156 Augsburg, Germany; dmytro.vlasenko@uk-augsburg.de; 3 Tumor Data Management, University Hospital Augsburg, 86156 Augsburg, Germany; Gerhard.Schenkirsch@uk-augsburg.de; 4 Institute of Mathematics, Augsburg University, 86156 Augsburg, Germany; stefan.schiele@math.uni-augsburg.de (S.S.); gernot.mueller@math.uni-augsburg.de (G.M.)

**Keywords:** colon cancer, Stroma AReactive Invasion Front Areas, SARIFA, biomarker, histopathology

## Abstract

**Simple Summary:**

Many studies have used histomorphological features to more precisely predict the prognosis of patients with colon cancer, focusing on tumor budding, poorly differentiated clusters, and the tumor–stroma ratio. Here, we introduce SARIFA: Stroma AReactive Invasion Front Area(s). We defined SARIFA as the direct contact between a tumor gland/tumor cell cluster (≥5 cells) and inconspicuous surrounding adipose tissue in the invasion front. SARIFA shows an excellent interobserver reliability and high prognostic value and is thus a promising histomorphological prognostic indicator for adipose-infiltrative adenocarcinomas of the colon.

**Abstract:**

Many studies have used histomorphological features to more precisely predict the prognosis of patients with colon cancer, focusing on tumor budding, poorly differentiated clusters, and the tumor–stroma ratio. Here, we introduce SARIFA: Stroma AReactive Invasion Front Area(s). We defined SARIFA as the direct contact between a tumor gland/tumor cell cluster (≥5 cells) and inconspicuous surrounding adipose tissue in the invasion front. In this retrospective, single-center study, we classified 449 adipose-infiltrative adenocarcinomas (not otherwise specified) from two groups based on SARIFA and found 25% of all tumors to be SARIFA-positive. Kappa values between the two pathologists were good/very good: 0.77 and 0.87. Patients with SARIFA-positive tumors had a significantly shorter colon-cancer-specific survival (*p* = 0.008, group A), absence of metastasis, and overall survival (*p* < 0.001, *p* = 0.003, group B). SARIFA was significantly associated with adverse features such as pT4 stage, lymph node metastasis, tumor budding, and higher tumor grade. Moreover, SARIFA was confirmed as an independent prognostic indicator for colon-cancer-specific survival (*p* = 0.011, group A). SARIFA assessment was very quick (<1 min). Because of low interobserver variability and good prognostic significance, SARIFA seems to be a promising histomorphological prognostic indicator in adipose-infiltrative adenocarcinomas of the colon. Further studies should validate our results and also determine whether SARIFA is a universal prognostic indicator in solid cancers.

## 1. Introduction

Colorectal cancer is a major public health problem worldwide. In 2018, colorectal cancer was estimated to cause 881,000 deaths [1]. The histopathological assessment of colorectal cancer is crucial to assess the prognosis and determine the therapeutic approach [2]. The established criteria include depth of infiltration/T stage, lymph node involvement, histological subtype, histological grade, differentiation, venous and lymphatic invasion, perineural invasion, and status of surgical resection margins [3]. Other histomorphological features associated with colorectal cancer include tumor border configuration (according to Jass/expansile versus indeterminate versus infiltrative) [4,5,6,7], tumor deposits [8,9], tumor budding [10,11,12,13,14,15], poorly differentiated clusters [16,17,18,19] and the tumor–stroma ratio [20,21,22,23,24,25,26,27]. It would certainly be beneficial to include some of the features in the routine diagnostics. However, defining their uniform criteria and reducing interobserver variability remain challenging for some parameters [7,15,28,29,30].

**Figure 1:**
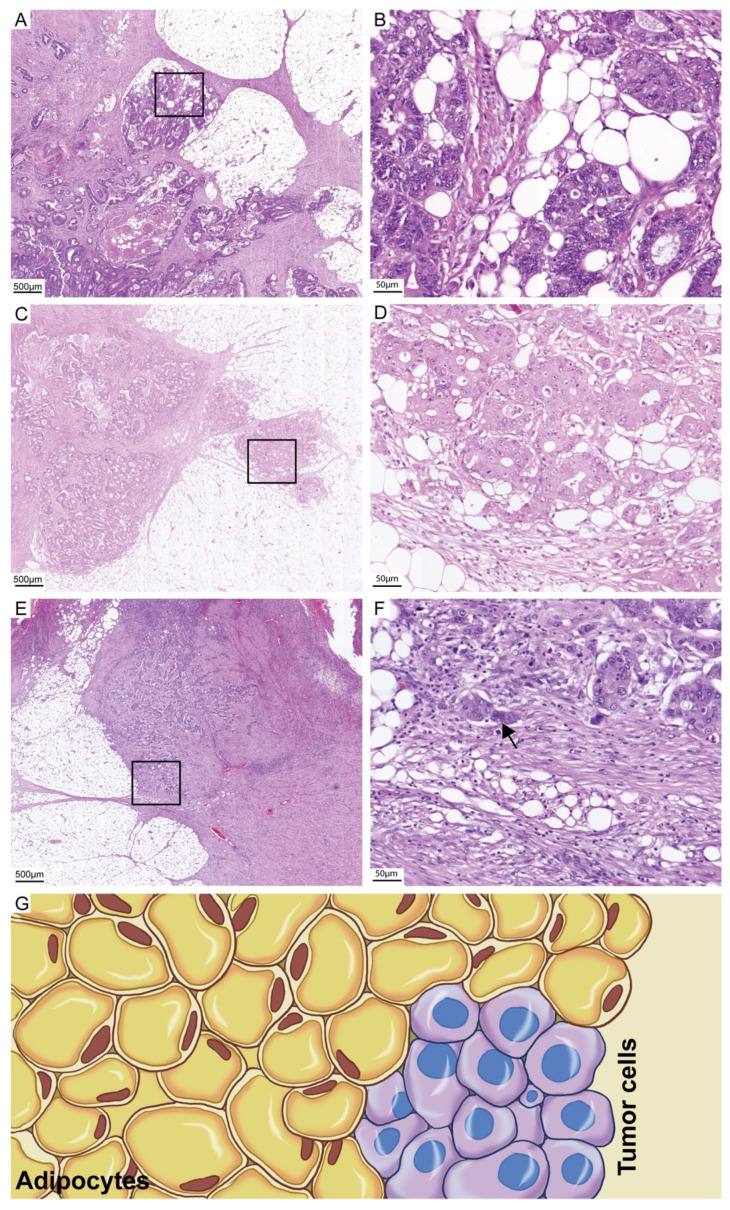
H & E images of SARIFA-positive and SARIFA-negative cases: SARIFA-positive cases (**A**,**C**: scale bar 500 µm; **B**,**D**: scale bar 50 µm) SARIFA-negative case at the invasion front with a PDC (arrow) (**E**: scale bar 500 µm, **F**; scale bar 50 µm). Schematic representation of a SARIFA area with tumor cells directly adjacent to adipocytes without a stromal reaction (**G**).

In this study, we investigated a specific histopathological characteristic, which we noticed during routine diagnostics. We observed that in cases with high-grade features, tumor cells are often completely unresponsive in the adipose tissue in the invasion front (Figure 1A–G).

We termed this as the stroma-areactive invasion front area (SARIFA). Wen et al. identified that adipocytes in the tumor microenvironment serve as energy providers and metabolic regulators to promote the growth and survival of colon cancer cells [31]. Therefore, we investigated the prognostic significance of SARIFA in adipose-infiltrative colon carcinomas and analyzed the interobserver variability.

We hypothesized that SARIFA is associated with high-grade features (e.g., vascular invasion) and is independent prognostic factor. First, we conducted an orientational analysis on 196 adipose-infiltrative adenocarcinomas (not otherwise specified, NOS) of the colon. Next, we analyzed a different group to substantiate the results.

## 2. Materials and Methods

### 2.1. Case Groups

This study conforms to the REMARK [32] and STROBE [33] guidelines. Patient group A comprised 196 consecutive patients with colon adenocarcinomas NOS, pT3/4, N+/−, M0, R0, and ≥3 months survival after surgery. Surgeries were performed between January 2002 and December 2011 at the University Medical Center of Augsburg, Germany. Patient group B comprised 253 consecutive patients with the same inclusion criteria, except that the surgeries were performed between 2012 and 2016. None of the patients had received preoperative chemo- or radiotherapy. After surgery, patients’ tumor tissue was fixed immediately in 4% buffered formalin for at least 12 h and then embedded in paraffin. The slides investigated were made of 3 µm FFPE tissue block sections. The Cannon–Böhm point was used to classify the sidedness (right versus left). Tumor budding was graded according to the criteria of the international tumor budding consensus conference (ITBCC). In group A, consensus rating was used to define the budding grade, as recently published [15]. In group B, tumor budding was assessed by a single investigator on the same slide on which SARIFA was assessed. The Tumor Data Management, University Hospital of Augsburg, provided the data on follow-up and adjuvant therapy, and these data were complemented with those of the patient files. In general, adjuvant therapy was conducted according to the guidelines in place at the time. The study protocol was approved by the Institutional Review Board of University Hospital of Augsburg (5.5.2020, BKF 2017-12, and 2020-24). A population size of approx. 250 patients for group B appeared to us to be sufficient in the study design due to a calculated power of approx. 75% (assuming the observed parameters in group A: SARIFA frequency 16%, colon cancer-specific death 26% versus 9%) [34]. Because SARIFA was evaluated only in one slide of each case, the question had to be answered in which slide based frequency SARIFA can be found. This was evaluated on a separate cohort (group C) from 2017. A restriction to nodal positive cases yielded in an enrichment of SARIFA-positive cases. Moreover, this cohort was used to evaluate the relation between SARIFA and five other morphological biomarkers (tumor budding, poorly differentiated clusters, stroma grading and infiltration classification according to Jass) [5,6,12,35,36].

### 2.2. Definition and Assessment of SARIFA and Other Morphological Biomarkers

SARIFA was evaluated based on entire slide sections that covered about 220 mm^2^. We have defined SARIFA as an area located at the invasion front (IF) in which a tumor gland or a group of at least five tumor cells directly approach adipocytes without a stromal reaction, such as fibroblastic proliferation, collagen formation, or a histiocytic reaction. We classified tumors that presented these characteristics as SARIFA-positive and the others as SARIFA-negative. Even if only a single SARIFA was present (e.g., a single tumor gland surrounded directly by inconspicuous adipose tissue), we classified the tumor as SARIFA-positive. The first author (FA) and the last author (LA) independently assessed all tumors. They were blinded to the results of each other, clinical data, and clinical course. A representative tumor slide (Group A: highest degree of tumor budding; Group B: greatest depth of infiltration) was used for assessment in each case. In the event of different results, the cases were discussed on a double head microscope, and a consensus diagnosis was made.

The biomarkers tumor budding, poorly differentiated clusters, stroma grading and infiltration classification according to Jass were analyzed by LA. All parameters besides the Jass classification were analyzed initially in three-tiered fashion and then transferred in two tired grading by merging the grades one and two to a low-grade group and the grade three cases to the high-grade group.

### 2.3. Statistical Analysis

All statistical analyses were performed using SPSS version 24.0 (SPSS, IBM, Chicago, IL, USA) and R Version 4.0.3. Depending on the size of the proportions, the χ^2^ and the Fisher Exact test were used to compare categorical data, and the Mann–Whitney U test was used for comparisons continuous and ordinal variables between the two groups. *p*-values were adjusted for multiple testing using the Bonferroni Holm method [37]. Kappa statistics were used to measure interobserver agreement (<0.2: poor, 0.21–0.40: fair, 0.40–0.60: moderate, 0.61–0.80: good, and >0.80 very good [38]). The Spearman rank order correlation was used to analyze the relation of ranked parameters. Kaplan–Meier estimates of survival rates were compared using log-rank tests, and relative risks were estimated by hazard ratios (HR) obtained from Cox proportional hazard models. Exploratory significance levels (two-tailed) of 5% were used for hypothesis testing. Continuous variables are presented as median with corresponding 25% and 75% quantile.

## 3. Results

### 3.1. Duration of the Assessment and Interobserver Variability

In most cases, SARIFA could be assessed very quickly. To estimate the time required, 40 cases were reassessed by the FA, and the time was measured. The minimum time was 13 s, and the maximum time was 40 s. The mean time was 21 ± 6 s. There was a discrepancy in the SARIFA assessment by the investigators in group A in 7 (4%) cases and in group B in 25 (10%) cases. The corresponding kappa values were good/very good: 0.87 and 0.77 (group B), respectively.

### 3.2. Clinicopathological Characteristics

In group A, the mean age of the 196 patients at the date of diagnosis was 70.1 ± 11.3 years, the mean follow-up time was 5.3 ± 3.5 years, and the median follow-up time was 6.4 years [39]. The patients’ detailed clinicopathological characteristics are presented in Table 1. Of the 196 cases, 31 (16%) tumors were classified as SARIFA-positive and 164 (84%) as SARIFA-negative. During the follow-up period, 81 (41%) patients died.

In group B, the mean age of the 253 patients at the date of diagnosis was 69.1 ± 11.3 years, the mean follow-up time was 3.6 ± 1.9 years, and the median follow-up time was 4.2 years. The patients’ detailed clinicopathological characteristics are presented in Table 2. Of the 253 cases, 79 (31%) tumors were classified as SARIFA-positive and 174 (69%) as SARIFA-negative. During the follow-up period, 68 (27%) patients died.

Group C comprised 49 nodal positive cases. The clinicopathological characteristics are summarized in Table S1.

### 3.3. Characteristics of SARIFA-Positive Adenocarcinomas

SARIFA-positive tumors were associated with histopathological indicators of an adverse prognosis. Table 1 and Table 2 present an overview for groups A and B. In group A, SARIFA-positive tumors were associated with more frequent lymph node metastasis, higher count of metastasized lymph nodes, tumor budding, and higher grades (*p* = 0.009, *p* = 0.002, *p* = 0.004, and *p* = 0.02, respectively).

In group B, SARIFA-positive tumors also had more frequent lymph node metastasis, higher count of metastasized lymph nodes, higher vascular and lymphatic vessel invasion, high grades, and higher T stage (*p* < 0.001, *p* < 0.001, *p* = 0.016, *p* < 0.001, *p* = 0.015, and *p* < 0.001, respectively). Furthermore, patients with SARIFA-positive tumors were more likely to be women (*p* = 0.020) who received adjuvant chemotherapy more often (47% vs. 30% *p* = 0.009).

### 3.4. Univariate Prognostic Analyses

**Figure 2:**
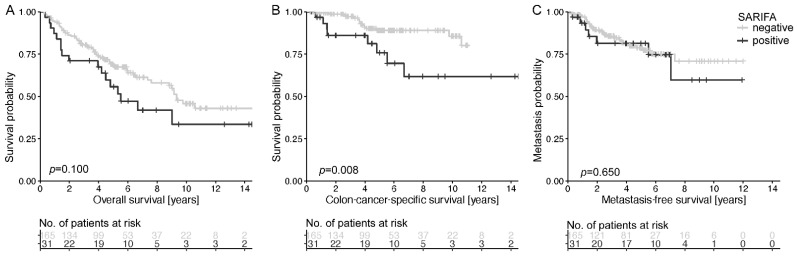
Discrimination of patient overall survival (**A**), colon-cancer-specific survival (**B**), and metastasis-free survival (**C**) based on SARIFA status in group A. The Kaplan–Meier curves of patients with SARIFA-positive and SARIFA-negative tumors are shown. *p*-value of log-rank test.

In group A, patients with SARIFA-positive tumors had a significantly shorter colon-cancer-specific survival (*p* = 0.008, Figure 2B). During the study period, 26% and 9% of patients with SARIFA-positive and SARIFA-negative tumors, respectively, died because of colon cancer (*p* = 0.100, Figure 2A). No significant association between SARIFA positivity and the occurrence of distant metastasis (*p* = 0.650, Figure 2B).

**Figure 3:**
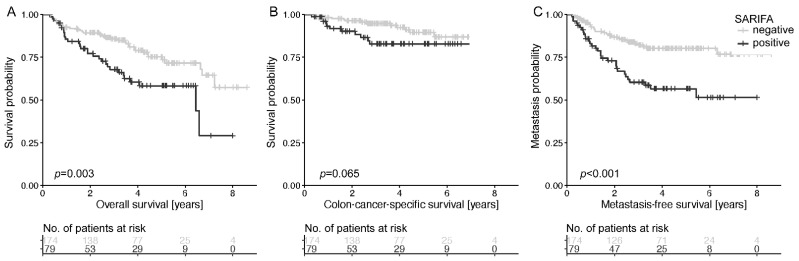
Discrimination of patient overall survival (**A**), colon-cancer-specific survival (**B**), and metastasis-free survival (**C**) based on SARIFA status in group B. The Kaplan–Meier curves of patients with SARIFA-positive and SARIFA-negative tumors are shown. *p*-value of log-rank test.

In group B, patients with SARIFA-positive tumors had a significantly shorter metastasis-free survival (*p* < 0.001) and shorter overall survival (*p* = 0.003) and tended to have a shorter colon-cancer-specific survival (*p* = 0.065, Figure 3A–C). The relative risk of dying or developing metastasis was about twice as high for patients with SARIFA-positive tumors (Table 1). The 5-year survival rate was 44% in patients with SARIFA-positive tumors and 63% in patients with SARIFA-negative tumors (*p* = 0.085). In subgroup analyses the negative prognostic effect of SARIFA was especially seen in stage III tumors (Table S3).

### 3.5. Multivariate Cox Regression

We performed multivariate Cox regression analyses (inclusion) for colon-cancer-specific survival (group A), overall survival (group B), and distant metastasis (group B). Each model was adjusted for the following parameters: age, pT, pN (positive versus negative), grading (low versus high), vascular and lymphatic vessel invasion, tumor budding, microsatellite stability status, sidedness, and SARIFA. The detailed results are summarized in Table 3. SARIFA was an independent prognostic indicator for colon-cancer-specific survival (*p* = 0.012, group A) after adjustment. The hazard ratio was 3.5 (CI 95%: 1.3–9.1). However, SARIFA could not be confirmed as an independent prognostic indicator for overall survival and distant metastasis (Table 3).

### 3.6. Analysis of SARIFA in T Stage Subgroups

To investigate the effect of SARIFA for both T stages (pT3 and pT4) separately, we conducted univariate and multivariate Cox regression analyses for overall survival and distant metastasis for group B in both subgroups. We adjusted for all variables as described above. In the pT3 stage, patients with SARIFA-positive tumors had a shorter absence of metastasis (*p* = 0.006) and a shorter overall-survival (*p* = 0.026). SARIFA was slightly not significant in both adjusted multivariate Cox regressions (metastasis: *p* = 0.058, overall-survival: *p* = 0.082). In comparison, we found no association between SARIFA and occurrence of metastasis (*p* = 0.132) or overall-survival (*p* = 0.193) in the higher T stage and SARIFA could not be shown to be an independent prognostic factor.

### 3.7. Slide-Based Frequency of SARIFA and Correlation with Other Morphological Biomarkers

Group C compromises a total number of 49 cases. From the 29 (59%) SARIFA-positive cases a total number of 91 histological tumor slides were available. SARIFA could be identified in 74 (76%) slides out of 91 slides. In 21 (72%) cases more than the half of the tumor slides were SARIFA-positive.

SARIFA showed a weak positive correlation with tumor budding and poorly differentiated clusters that just failed significancy with correlation coefficients of 0.267 (*p* = 0.063) and 0.281 (*p* = 0.050), respectively. Stroma grading and Jass’ infiltration grading showed no correlation. A strong correlation could be shown between tumor budding and poorly differentiated clusters (0.710; *p* < 0.0001). All calculations are given Table S2 (supplement). Simultaneous high-grade classification of the other morphological markers in SARIFA-positive cases occurred in a range between 28% (stroma grading) and 52% (Tumor budding) (Figure S1).

## 4. Discussion

In this study, we introduce a new biomarker in colon cancer—SARIFA (Figure 1). SARIFA has excellent interobserver reliability and high prognostic value and is thus a promising histomorphological prognostic indicator for adipose-infiltrative adenocarcinomas (NOS) of the colon. Patients with SARIFA-positive tumors had a significantly adverse prognosis: they had a significantly shorter colon-cancer-specific survival (*p* = 0.008, group A), absence of metastasis (*p* < 0.001, group B), and overall survival (*p* = 0.003, group B).

These results are particularly promising, given the low interobserver variability (kappa: 0.87 and 0.77 in groups A and B, respectively), which was likely due to the simple and clear definition of SARIFA-positive tumors—a single tumor gland/tumor cell cluster (≥5 cells) surrounded directly by inconspicuous adipose tissue. With this definition, no quantification of the characteristic is required (which may increase the interobserver variability). In our experience, the SARIFareas were usually considerably larger than one gland/ tumor cell cluster (≥5 cells) (see Figure 1) and therefore easy to assess. Among other things, we have used this definition to achieve an absolutely clear differentiation from tumor budding. SARIFA could be assessed with hardly any additional cost in routine diagnostics. The assessment is made on a standard hematoxylin–eosin slide from routine diagnostics and takes very little time. In most cases, SARIFA can be assessed extremely quickly (mean duration: 21 ± 6 s). Analyzing group C, we identified an uneven distribution of SARIFA areas among the analyzed slides of SARIFA positive cases. In the majority of cases more than a half of tumor slides are diagnostic for SARIFA positivity. Whether an increase in the number of examined slides leads to an improvement in sensitivity has to be clarified in further studies. If this were to be confirmed, the effort would nevertheless be limited due to the simplicity of the evaluation.

Currently, there are no uniform, established classification criteria for assessing the tumor border configuration at the invasion front. Depending on the definition, 17–70% of the tumors show an infiltrative growth [7,40,41]. Investigations of the tumor border configuration (as defined by Jass) indicated suboptimal interobserver agreement [6]. Tumor budding might also have considerable interobserver variability [15,29,42,43]. However, the tumor–stroma ratio indicated a similarly low interobserver variability as SARIFA, and the assessment was also relatively simple and quick (<2 min) [26]. In addition, a high reproducibility was reported for PDC grade [44]. SARIFA thus stands out in terms of interobserver variability, as PDC grade, and tumor–stroma ratio.

SARIFA may also allow risk stratification of patients with colon cancer. The hazard ratio for colon-cancer-specific survival was 3.5 (CI 95%: 1.3–9.1, *p* = 0.011, group A) after adjustment for age, pN, pT, grading, L, V, tumor budding, sidedness, and microsatellite status. Despite the numerous associations of SARIFA with a poor prognosis, significant association of SARIFA with colon-cancer-specific survival (*p* = 0.008) could be solely demonstrated in group A, whereas group B only exhibited a trend toward significance (*p* = 0.065). SARIFA was an independent risk factor only in group A. These differences are likely due to the differences between the two groups. A very likely main point is that the follow-up was relatively short in group B, with a mean follow-up of 3.6 ± 1.9 years. Furthermore, there was a lower proportion of pT3 tumors in group B. In the pT3 subgroup analysis of group B, SARIFA was a highly significant prognostic marker in the univariate analysis and just failed significance in the multivariate analysis (metastasis: *p* = 0.058, overall-survival: *p* = 0.082). Prospective large-scale studies with longer follow-up are needed for more consistent results. There was also a difference in the frequency of SARIFA-positive tumors between the two groups (16% versus 31%). All adenocarcinomas (NOS) were consecutively included according to the same criteria, and they were processed by the same institute, so differences in assessment become unlikely. Furthermore, FA assessed both groups simultaneously (mixed), ruling out the assessment order as a cause of the discrepancy. SARIFA-positive tumors had significantly more frequent histopathological features that were associated with an adverse prognosis, such as pT4 stage, lymph node metastasis, vascular and lymphatic vessel invasion, higher grade (*p* < 0.001, *p* < 0.001, *p* = 0.016, *p* < 0.001, and *p* = 0.015, respectively, group B), tumor budding (*p* < 0.001 (group B), *p* = 0.004 (group A)), and higher grade (*p* = 0.004, *p* = 0.02, group A), respectively. Therefore, further studies should consider these important features and elucidate the relationship between them.

Our study limitations include the retrospective single-center design of our study. Notably, however, Wulczyn et al. recently identified a similar pattern in colorectal cancer to be prognostic by using deep learning [45]. They identified that poorly differentiated tumor cell clusters adjacent to adipose tissue (TAF) can predict disease-specific survival for stage II-III colorectal cancer. Their results agree with ours. Most of their TAF examples in Figure S10 are SARIFAs. However, some of their presented cases were ambiguous, and some clearly had no SARIFAs (see representative images of Wulczyn et al. S10: no SARIFAS: first row, first image and last row, second, and third image). Considering the literature and our data, the tumor cells in the vicinity of adipose tissue may be of previously underestimated prognostic relevance.

Regarding the relation between SARIFA and other morphological markers like tumor budding, poorly differentiated clusters, stroma grading and infiltration classification according to Jass [5,6,12,35,36] we identified no or only weak correlations (tumor budding and poorly differentiated clusters). On the other hand, the latter correlated strongly with each other and reflect very likely the same biological background. Although SARIFA is found simultaneously with one or more of these five analyzed morphological features, we consider it highly probable that it is an expression of a biological property of its own. Jass’ classification might be an exemption in this regard because he described SARIFA-similar features in his classification before. Nevertheless, we found no relevant correlation between these parameters. Moreover, the Jass classification was hampered by a poor reproducibility [6].

From a biological point of view, it seems logical that SARIFA can have prognostic significance. Koelzer et al. stated that the invasion front of colorectal cancer embodies the tumor–host interface [14]. The interaction between tumor cells and adipose tissue received an increasing interest in the last ten years outgoing from the fact that obesity is associated with both increased rates of malignancy and increased aggressiveness of tumor diseases. This rapidly expanding field is particularly relevant for future therapy strategies including the recognition of resistance mechanisms and new targets [46]. SARIFA has a distinct feature of this tumor–host interface with striking morphology. Currently unknown variables of the tumor, tumor microenvironment, or even their interaction of both might lead to this characteristic morphology. Adipocytes can serve as energy providers and metabolic regulators to promote the growth and survival of colon cancer cells [31], but this explanation does not sufficiently explain the prognostic effect observed here. We observed increased CD68-positive macrophages at the invasion front of SARIFA tumors. It is, therefore, probable that macrophages play a role in preventing the tumor glands from being entrapped by desmoplastic fibers and induce a reduced response of the immune system in SARIFA-tumors [47]. We therefore think that tumor-associated macrophages play a role here, but this requires investigation in further studies. This study explored the prognostic significance of SARIFA. However, further studies are required to investigate the details of the underlying molecular biological background as well as potential associations to other histopathological prognostic indicators (for example, stroma differentiation grading, PDC grade and tumor–stroma ratio), which have not been fully determined. Independent of colon carcinomas, whether SARIFA is a universal feature of malignancy remains unclear. After tumor budding was established as prognostic parameter in colorectal carcinomas, it was also demonstrated in numerous solid cancers [48]. In general, tumor budding is primarily interpreted as an expression of epithelial–mesenchymal transition (EMT) [49,50,51], whereby the concept of EMT is also controversially discussed [52]. Future studies should validate our results to better characterize the importance of SARIFA.

## 5. Conclusions

We introduced a new biomarker in colon cancer: the Stroma AReactive Invasion Front Area (SARIFA). Our results provide first evidence that SARIFA is a promising histomorphological prognostic indicator in adipose-infiltrative adenocarcinomas (NOS) of the colon. Further research is warranted to examine whether SARIFA can serve as a prognostic indicator in solid cancers as well.

## Figures and Tables

**Table 1:** Clinicopathological characteristics group A.

Variable		*n* = 196		SARIFA-Positive (*n* = 31)		SARIFA-Negative (*n* = 165)		*p*-Value/*p*-Value Adjusted *
Median Age [years]		71 (64–79)		70 (64–74)		72(64–79)		0.129/1.0
Median Follow-up (95% CI) [years]		6.4 (5.6–7.1)		6.0 (5.5–6.6)		7.0 (5.0–9.1)		0.581/1.0
Median Lymph Node Harvest (*n*)		20 (13–30)		20 (15–40)		19 (13–29)		0.254/1.0
Positive Lymph Nodes (*n*)		0 (0–1)		1 (0–4)		0 (0–1)		0.002/0.036
Sex								0.843/1.0
	female	83	42%	14	45%	69	42%	
	male	113	58%	17	55%	96	58%	
T status								0.229/1.0
	pT3	172	88%	25	81%	147	89%	
	pT4	24	12%	6	19%	18	11%	
N status								0.009/0.144
	negative	119	61%	12	39%	107	65%	
	positive	77	39%	19	61%	58	35%	
Grading								0.020/0.280
	low grade	137	70%	16	52%	121	73%	
	high grade	59	30%	15	48%	44	27%	
Vascular Invasion								0.098/1.0
	negative	176	90%	25	81%	151	91%	
	positive	20	10%	6	19%	14	9%	
Lymphatic Vessel Invasion								0.602/1.0
	negative	164	84%	25	81%	139	84%	
	positive	32	16%	6	19%	26	16%	
Tumor Budding								0.004/0.068
	Bd1	161	82%	20	65%	141	86%	
	Bd2	23	12%	6	19%	17	10%	
	Bd3	12	6%	5	16%	7	4%	
Location								0.841/1.0
	right sided	120	61%	20	65%	100	61%	
	left sided	76	39%	11	36%	65	39%	
MSS								0.773/1.0
	stable	172	88%	28	90%	144	87%	
	instable	24	12%	3	10%	21	13%	
Adjuvant Chemotherapy								0.123/1.0
	no	107	55%	13	42%	94	57%	
	yes	89	45%	18	58%	71	43%	
Distant Metastasis								0.624/1.0
	no	158	81%	24	77%	134	81%	
	yes	38	19%	7	23%	31	19%	
Death								0.113/1.0
	no	115	59%	14	45%	101	61%	
	death	81	41%	17	55%	64	39%	
Colon-Cancer-Specific Survival								0.014/0.210
	no	173	88%	23	74%	150	91%	
	death	23	12%	8	26%	15	9%	
Five Year Survival (*n* = 152)								0.272/1.0
	survived	92	60%	13	50%	79	63%	
	death	60	40%	13	50%	47	37%	

Legend: 95% CI, 95% confidence interval; *p*-values are shown for difference between SARIFA (Stroma AReactive Invasion Front Areas) positive and SARIFA-negative tumors; Abbreviations: MSS: microsatellite stability status; * adjusted using Bonferroni Holm method [37].

**Table 2:** Clinicopathological characteristics group B.

Variable		*n* = 253		SARIFA-Positive (*n* = 79)		SARIFA-Negative (*n* = 174)		*p*-Value *
Median Age [years]		71 (61–77)		72 (61–78)		71 (61–77)		0.658/1.0
Median Follow-up [years]		4.2 (4.0–4.4)		4.2 (3.9–4.5)		4.2 (3.7–4.7)		0.094/0.595
Median Lymph Node Harvest (*n*)		38 (29–49)		36 (28–53)		39 (30–49)		0.695/1.0
Positive Lymph Nodes (*n*)		0 (0–2)		1 (0–4)		0 (0–1)		<0.001/0.009
Sex								0.02/0.160
	female	110	44%	43	54%	67	39%	
	male	143	57%	36	46%	107	62%	
T status								<0.001/0.009
	pT3	179	71%	43	54%	136	78%	
	pT4	74	29%	36	46%	38	22%	
N status								<0.001/0.009
	negative	144	57%	25	32%	119	68%	
	positive	109	43%	54	68%	55	32%	
Grading								0.015/0.150
	low grade	213	84%	60	76%	153	88%	
	high grade	40	16%	19	24%	21	12%	
Vascular Invasion								0.016/0.150
	negative	218	86%	62	79%	156	90%	
	positive	35	14%	17	21%	18	10%	
Lymphatic Vessel Invasion								<0.001/0.009
	negative	197	78%	50	63%	147	85%	
	positive	56	22%	29	37%	27	16%	
Tumor Budding								<0.001/0.009
	Bd1	161	63%	33	42%	128	74%	
	Bd2	50	20%	27	34%	23	13%	
	Bd3	42	17%	19	24%	23	13%	
Location								0.785/1.0
	right	139	55%	42	53%	97	56%	
	left	114	45%	37	47%	77	44%	
MSS								0.580/1.0
	stable	213	84%	68	86%	145	83%	
	instable	40	16%	11	14%	29	17%	
Adjuvant Chemotherapy								0.009/0.108
	no	164	65%	42	53%	122	70%	
	yes	89	35%	37	47%	52	30%	
Distant Metastasis								<0.001/0.009
	no	192	76%	48	61%	144	83%	
	yes	61	24%	31	39%	30	17%	
Death								0.009/0.108
	no	185	73%	49	62%	136	78%	
	death	68	27%	30	38%	38	22%	
Colon-Cancer-Specific Survival								0.105/0.595
	no	229	90%	68	86%	161	92%	
	death	24	10%	11	14%	13	8%	
Five Year Survival (*n* = 117)								0..085/0.595
	survived	65	56%	20	44%	45	63%	
	death	52	44%	25	56%	27	38%	

Legend: 95% CI, 95% confidence interval; *p*-values are shown for difference between SARIFA (Stroma AReactive Invasion Front Areas) positive and SARIFA-negative tumors; Abbreviations: MSS: microsatellite stability status; * adjusted using Bonferroni Holm method [37].

**Table 3:** Cox regression analyses.

Variation	Group A (*n* = 196)			Group B (*n* = 253)					
	Colon-Cancer-Specific Survival			Metastasis			Overall Survival		
	HR	CI	*p*	HR	CI	*p*	HR	CI	*p*
T-status	1.4	0.4–5.0	0.57	2.1	1.9–3.6	0.01	1.4	0.8–2.4	0.20
N-status	1.8	0.7–4.6	0.26	3.1	1.6–5.7	<0.001	2.0	1.2–3.5	0.01
Age	1.1	1.0–1.1	0.02	1.0	0.9–1.0	0.15	1.05	1.0–1.1	<0.001
V	0.9	0.3–3.2	0.89	1.0	0.5–2.0	0.99	0.9	0.4–1.7	0.65
L	1.3	0.4–4.1	0.65	1.6	0.9–2.8	0.14	1.5	0.9–2.7	0.13
Grading	0.9	0.4–2.2	0.82	1.5	0.8–3.0	0.23	2.0	1.0–3.8	0.04
Tumor Budding	0.9	0.5–1.9	0.88	0.9	0.7–1.3	0.59	1.1	0.8–1.5	0.71
Location	1.9	0.8–4.5	0.14	0.9	0.5–1.5	0.64	1.2	0.7–2.0	0.52
MSS	0.3	0.0–2.6	0.29	0.7	0.3–1.6	0.39	0.7	0.3–1.5	0.40
SARIFA	3.5	1.3–9.1	0.01	1.5	0.8–2.6	0.18	1.5	0.9–2.5	0.14

Legend: CI: Confidence interval (95%), MSS: microsatellite stability status.

## Data Availability

The datasets generated during and/or analyzed during the current study are available from the corresponding author on reasonable request.

## Supplementary Materials

The following are available online at https://www.mdpi.com/article/10.3390/cancers13194880/s1, Figure S1: Percentage of high-grade features of morphological biomarkers depending on the SARIFA grading in group C., Table S1: Clinicopathological characteristics of group C., Table S2: Spearman Correlation of morphological biomarkers., Table S3. Subgroup analyses of SARIFA in UICC stage II vs. III.

## References

[1] Bray F., Ferlay J., Soerjomataram I., Siegel R.L., Torre L.A., Jemal A. (2018). Global cancer statistics 2018: GLOBOCAN estimates of incidence and mortality worldwide for 36 cancers in 185 countries. CA Cancer J. Clin..

[2] Fleming M., Ravula S., Tatishchev S.F., Wang H.L. (2012). Colorectal carcinoma: Pathologic aspects. J. Gastrointest. Oncol..

[3] Marzouk O., Schofield J. (2011). Review of histopathological and molecular prognostic features in colorectal cancer. Cancers.

[4] Jass J.R., Atkin W.S., Cuzick J., Bussey H.J., Morson B.C., Northover J.M., Todd I.P. (1986). The grading of rectal cancer: Historical perspectives and a multivariate analysis of 447 cases. Histopathology.

[5] Jass J.R., Love S.B., Northover J.M. (1987). A new prognostic classification of rectal cancer. Lancet.

[6] Jass J.R., Ajioka Y., Allen J.P., Chan Y.F., Cohen R.J., Nixon J.M., Radojkovic M., Restall A.P., Stables S.R., Zwi L.J. (1996). Assessment of invasive growth pattern and lymphocytic infiltration in colorectal cancer. Histopathology.

[7] Koelzer V.H., Lugli A. (2014). The tumor border configuration of colorectal cancer as a histomorphological prognostic indicator. Front. Oncol..

[8] Jin M., Roth R., Rock J.B., Washington M.K., Lehman A., Frankel W.L. (2015). The impact of tumor deposits on colonic adenocarcinoma AJCC TNM staging and outcome. Am. J. Surg. Pathol..

[9] Basnet S., Lou Q.F., Liu N., Rana R., Shah A., Khadka M., Warrier H., Sigdel S., Dhakal S., Devkota A. (2018). Tumor deposit is an independent prognostic indicator in patients who underwent radical resection for colorectal cancer. J. Cancer.

[10] Hase K., Shatney C., Johnson D., Trollope M., Vierra M. (1993). Prognostic value of tumor “budding” in patients with colorectal cancer. Dis. Colon. Rectum.

[11] Ueno H., Murphy J., Jass J., Mochizuki H., Talbot I. (2002). Tumourbudding’as an index to estimate the potential of aggressiveness in rectal cancer. Histopathology.

[12] Lugli A., Kirsch R., Ajioka Y., Bosman F., Cathomas G., Dawson H., El Zimaity H., Flejou J.F., Hansen T.P., Hartmann A. (2017). Recommendations for reporting tumor budding in colorectal cancer based on the International Tumor Budding Consensus Conference (ITBCC) 2016. Mod. Pathol..

[13] Mitrovic B., Schaeffer D.F., Riddell R.H., Kirsch R. (2012). Tumor budding in colorectal carcinoma: Time to take notice. Mod. Pathol..

[14] Koelzer V.H., Zlobec I., Lugli A. (2016). Tumor budding in colorectal cancer—Ready for diagnostic practice?. Hum. Pathol..

[15] Martin B., Schafer E., Jakubowicz E., Mayr P., Ihringer R., Anthuber M., Schenkirsch G., Schaller T., Markl B. (2018). Interobserver variability in the H&E-based assessment of tumor budding in pT3/4 colon cancer: Does it affect the prognostic relevance?. Virchows Arch..

[16] Kinoshita O., Kishimoto M., Murayama Y., Kuriu Y., Nakanishi M., Sakakura C., Otsuji E., Yanagisawa A. (2016). The number of metastatic lymph nodes exhibiting poorly differentiated clusters predicts survival in patients with pStage III colorectal cancer. Int. J. Colorectal Dis..

[17] Barresi V., Branca G., Vitarelli E., Tuccari G. (2014). Micropapillary pattern and poorly differentiated clusters represent the same biological phenomenon in colorectal cancer: A proposal for a change in terminology. Am. J. Clin. Pathol..

[18] Bonetti L.R., Barresi V., Bettelli S., Domati F., Palmiere C. (2016). Poorly differentiated clusters (PDC) in colorectal cancer: What is and ought to be known. Diagn. Pathol..

[19] Ueno H., Kajiwara Y., Shimazaki H., Shinto E., Hashiguchi Y., Nakanishi K., Maekawa K., Katsurada Y., Nakamura T., Mochizuki H. (2012). New criteria for histologic grading of colorectal cancer. Am. J. Surg. Pathol..

[20] Park J., Richards C., McMillan D., Horgan P., Roxburgh C. (2014). The relationship between tumour stroma percentage, the tumour microenvironment and survival in patients with primary operable colorectal cancer. Ann. Oncol..

[21] Mesker W.E., Junggeburt J., Szuhai K., de Heer P., Morreau H., Tanke H.J., Tollenaar R.A. (2007). The carcinoma–stromal ratio of colon carcinoma is an independent factor for survival compared to lymph node status and tumor stage. Anal. Cell. Pathol..

[22] Mesker W.E., Liefers G.J., Junggeburt J.M., van Pelt G.W., Alberici P., Kuppen P.J., Miranda N.F., van Leeuwen K.A., Morreau H., Szuhai K. (2009). Presence of a high amount of stroma and downregulation of SMAD4 predict for worse survival for stage I-II colon cancer patients. Cell. Oncol..

[23] West N., Dattani M., McShane P., Hutchins G., Grabsch J., Mueller W., Treanor D., Quirke P., Grabsch H. (2010). The proportion of tumour cells is an independent predictor for survival in colorectal cancer patients. Br. J. Cancer.

[24] Hynes S.O., Coleman H.G., Kelly P.J., Irwin S., O’Neill R.F., Gray R.T., McGready C., Dunne P.D., McQuaid S., James J.A. (2017). Back to the future: Routine morphological assessment of the tumour microenvironment is prognostic in stage II/III colon cancer in a large population-based study. Histopathology.

[25] Eriksen A.C., Sørensen F.B., Lindebjerg J., Hager H., Christensen R.d., Kjær-Frifeldt S., Hansen T.F. (2018). The prognostic value of tumour stroma ratio and tumour budding in stage II colon cancer. A nationwide population-based study. Int. J. Colorectal Dis..

[26] Van Pelt G.W., Kjaer-Frifeldt S., van Krieken J., Al Dieri R., Morreau H., Tollenaar R., Sorensen F.B., Mesker W.E. (2018). Scoring the tumor-stroma ratio in colon cancer: Procedure and recommendations. Virchows Arch..

[27] Martin B., Banner B.M., Schafer E.M., Mayr P., Anthuber M., Schenkirsch G., Markl B. (2020). Tumor proportion in colon cancer: Results from a semiautomatic image analysis approach. Virchows Arch..

[28] Bokhorst J., Blank A., Lugli A., Zlobec I., Dawson H., Vieth M., Rijstenberg L., Brockmoeller S., Urbanowicz M., Flejou J. (2020). Assessment of individual tumor buds using keratin immunohistochemistry: Moderate interobserver agreement suggests a role for machine learning. Mod. Pathol..

[29] Hacking S., Angert M., Jin C., Kline M., Gupta N., Cho M., Thomas R., Lee L., Chavarria H., Nasim M. (2019). Tumor budding in colorectal carcinoma: An institutional interobserver reliability and prognostic study of colorectal adenocarcinoma cases. Ann. Diagn. Pathol..

[30] Kai K., Aishima S., Aoki S., Takase Y., Uchihashi K., Masuda M., Nishijima-Matsunobu A., Yamamoto M., Ide K., Nakayama A. (2016). Cytokeratin immunohistochemistry improves interobserver variability between unskilled pathologists in the evaluation of tumor budding in T1 colorectal cancer. Pathol. Int..

[31] Wen Y.A., Xing X., Harris J.W., Zaytseva Y.Y., Mitov M.I., Napier D.L., Weiss H.L., Mark Evers B., Gao T. (2017). Adipocytes activate mitochondrial fatty acid oxidation and autophagy to promote tumor growth in colon cancer. Cell Death Dis..

[32] Altman D.G., McShane L.M., Sauerbrei W., Taube S.E. (2012). Reporting recommendations for tumor marker prognostic studies (REMARK): Explanation and elaboration. BMC Med..

[33] Von Elm E., Altman D.G., Egger M., Pocock S.J., Gotzsche P.C., Vandenbroucke J.P., Initiative S. (2007). The Strengthening the Reporting of Observational Studies in Epidemiology (STROBE) statement: Guidelines for reporting observational studies. Lancet.

[34] Faul F., Erdfelder E., Lang A.G., Buchner A. (2007). G*Power 3: A flexible statistical power analysis program for the social, behavioral, and biomedical sciences. Behav. Res. Methods.

[35] Ueno H., Hase K., Hashiguchi Y., Shimazaki H., Tanaka M., Miyake O., Masaki T., Shimada Y., Kinugasa Y., Mori Y. (2014). Site-specific tumor grading system in colorectal cancer: Multicenter pathologic review of the value of quantifying poorly differentiated clusters. Am. J. Surg. Pathol..

[36] Hacking S.M., Chakraborty B., Nasim R., Vitkovski T., Thomas R. (2021). A Holistic Appraisal of Stromal Differentiation in Colorectal Cancer: Biology, Histopathology, Computation, and Genomics. Pathol. Res. Pract..

[37] Holm S. (1979). A simple sequentially rejective multiple test procedure. Scand. J. Stat..

[38] Landis J.R., Koch G.G. (1977). The measurement of observer agreement for categorical data. Biometrics.

[39] Schemper M., Smith T.L. (1996). A note on quantifying follow-up in studies of failure time. Control. Clin. Trials.

[40] Zlobec I., Baker K., Minoo P., Hayashi S., Terracciano L., Lugli A. (2009). Tumor border configuration added to TNM staging better stratifies stage II colorectal cancer patients into prognostic subgroups. Cancer.

[41] Kubota Y., Petras R.E., Easley K.A., Bauer T.W., Tubbs R.R., Fazio V.W. (1992). Ki-67-determined growth fraction versus standard staging and grading parameters in colorectal carcinoma. A multivariate analysis. Cancer.

[42] Martin B., Mayr P., Ihringer R., Schafer E.M., Jakubowicz E., Anthuber M., Schenkirsch G., Schaller T., Markl B. (2020). Interobserver Variability in the Assessment of Tumor Budding in pT 3/4 Colon Cancer: Improvement by Supporting Immunohistochemistry?. Diagnostics.

[43] Puppa G., Senore C., Sheahan K., Vieth M., Lugli A., Zlobec I., Pecori S., Wang L.M., Langner C., Mitomi H. (2012). Diagnostic reproducibility of tumour budding in colorectal cancer: A multicentre, multinational study using virtual microscopy. Histopathology.

[44] Shivji S., Conner J.R., Barresi V., Kirsch R. (2020). Poorly differentiated clusters in colorectal cancer: A current review and implications for future practice. Histopathology.

[45] Wulczyn E., Steiner D.F., Moran M., Plass M., Reihs R., Tan F., Flament-Auvigne I., Brown T., Regitnig P., Chen P.C. (2021). Interpretable survival prediction for colorectal cancer using deep learning. NPJ Digit. Med..

[46] Lengyel E., Makowski L., DiGiovanni J., Kolonin M.G. (2018). Cancer as a matter of fat: The crosstalk between adipose tissue and tumors. Trends Cancer.

[47] Zhou J., Tang Z., Gao S., Li C., Feng Y., Zhou X. (2020). Tumor-Associated Macrophages: Recent Insights and Therapies. Front. Oncol..

[48] Berg K.B., Schaeffer D.F. (2018). Tumor budding as a standardized parameter in gastrointestinal carcinomas: More than just the colon. Mod. Pathol..

[49] Lugli A., Zlobec I., Berger M.D., Kirsch R., Nagtegaal I.D. (2021). Tumour budding in solid cancers. Nat. Rev. Clin. Oncol..

[50] Zlobec I., Lugli A. (2010). Epithelial mesenchymal transition and tumor budding in aggressive colorectal cancer: Tumor budding as oncotarget. Oncotarget.

[51] Grigore A.D., Jolly M.K., Jia D., Farach-Carson M.C., Levine H. (2016). Tumor Budding: The Name is EMT. Partial EMT. J. Clin. Med..

[52] Tarin D., Thompson E.W., Newgreen D.F. (2005). The fallacy of epithelial mesenchymal transition in neoplasia. Cancer Res..

