# Supplementary material for: Stroma AReactive Invasion Front Areas (SARIFA)—A New Easily to Determine Biomarker in Colon Cancer—Results of a Retrospective Study
Source: Cancers (Basel). 2021 Sep 29;13(19):4880. doi: 10.3390/cancers13194880 (PMC8508517; doi:10.3390/cancers13194880)
Supplement: Supplementary file 1 [file cancers-13-04880-s001.zip › cancers-1374890-supplementary.pdf]

Table S1. Clinicopathological characteristics of group C

**Variable**		***n* = 49**	**SARIFA-positive (*n* = 29)**	**SARIFA-negative (*n* = 20)**	**P-value**
Median Age (years)		71 (61-77)	72 (61-78)	71 (61-77)	0.458
MedianLymph Node Harvest (n)		38 (29-49)	36 (28-53)	39 (30-49)	0.776
MedianPositive Lymph Nodes (n)		0 (0-2)	1 (0-4)	0 (0-1)	0.992
Sex					0.842
	female	20 (41%)	12 (41%)	8 (40%)	
	male	29 (59%)	17 (59%)	12 (60%)	
T status					0.042
	pT3	35 (71%)	12 (41%)	15 (75%)	
	pT4	14 (29%)	17 (59%)	5 (25%)	
Grading					0.700
	low grade	41 (84%)	25 (86%)	16 (80%)	
	high grade	8 (16%)	4 (14%)	4 (20%)	
Vascular invasion					0.234
	negative	36 (73%)	19 (66%)	17 (85%)	
	positive	13 (27%)	10 (34%)	3 (15%)	
Lymphatic invasion					0.512
	negative	37 (76%)	23 (79%)	14 (70%)	
	positive	12 (24%)	6 (21%)	6 (30%)	
Location					0.842
	right	28 (57%)	12 (41%)	8 (40%)	
	left	21 (43%)	17 (59%)	12 (60%)	
MMS					1.000
	stabe	40 (95%)*	25 (96%)	16 (94%)	
	instable	2 (5%)*	1 (4%)	1 (6%)	
Adjuvant Therapy					0.272
	no	23 (47%)	13 (45%)	13 (65%)	
	yes	26 (53%)	16 (55%)	7 (35%)	
Death					0.496
	no	39 (80%)	22 (76%)	13 (65%)	
	yes	10 (20%)	7 (24%)	3 (15%)	

Table S2. Spearman Corelation of morphological biomarkers

	**Tumor budding**	**PDC**	**SG**	**Jass**
**SARIFA**	0.267	0.281	0.148	0.217
	0.0633	0.0503	0,308	0.134
	49	49	49	49
**Tumor budding**		0,710	0.250	0.253
		0.000000200	0.0832	0.0788
		49	49	49
**PDC**			0.0671	0.102
			0.645	0.482
			49	49

**SG**	0.231
	0.110
	49
**Jass**	

Legend: SARIFA, Stroma AReactive Invasion Front Areas; PDC, poorly differentiated clusters; SG, Stroma Grading ; Jass, Infiltration Classifiaction according to Jass

Table S3. Subgroup analyses of SARIFA in UICC stage II vs. III

		Group A + B			
**Overall survival**		HR	95%CI	p value	p value (log rank)
	Stage II (n=340)	1.299	0.696 – 2.424	0.411	0.409
	Stage III (n=140)	1.707	1.083 – 2.692	0.021	0.020
**Colon-cancer-specific survival**					
	Stage II (n=340)	0.832	0.188 – 3.675	0.808	0.808
	Stage III (n=140)	2.432	1.196 – 4.948	0.014	0.011
**Metastasis-free survival**					
	Stage II (n=340)	1.172	0.487-2.817	0.724	0.723
	Stage III (n=140)	1.916	1.168-3.144	0.010	0.009

HR: Hazard Ratio of SARIFA positive cases - Reference: SARIFA negative cases; CI: Confidence intervall (95%)

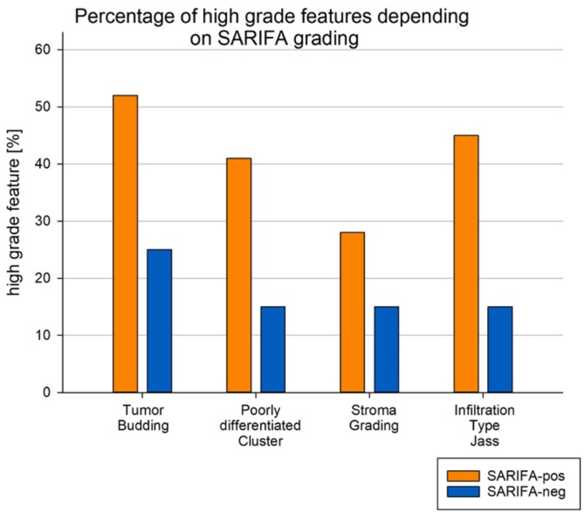

Figure S1 Percentage of high-grade features of morphological biomarkers depending on the SRAIFA grading in group C
